# Development and Application of an RPA-Based Rapid Point-of-Care Testing (POCT) Method for the Detection of Feline Panleukopenia Virus

**DOI:** 10.1155/2024/3680778

**Published:** 2024-08-24

**Authors:** Liang Hong, Qian Huang, Yuhang Zhou, Qi Zheng, Shipeng Wang, Fangfang Chen, Xinyue Chang, Guosheng Jiang, Lisha Zha

**Affiliations:** ^1^ International Immunology Centre College of Animal Science and Technology Anhui Agricultural University, Hefei 230036, Anhui, China; ^2^ Institute of Basic Medicine Shandong Academy of Medical Sciences, Jinan 250062, Shandong, China; ^3^ School of Biomedical Sciences Hunan University, Changsha 410000, Hunan, China

## Abstract

Feline panleukopenia (FP) is a highly prevalent and consequential disease that poses a substantial threat to both adult and juvenile cats across all geographical regions. The causative agent responsible for this disease is the feline panleukopenia virus (FPV). Therefore, it is imperative to develop a facile, efficient, and accurate detection method for FPV. Hence, a recombinase polymerase amplification–lateral flow dipstick assay (RPA–LFDA) method was specifically designed for the detection of FPV. The amplification process was optimized. This investigation focused on evaluating the expansion temperature detection system and revealed an optimal reaction temperature of 39°C. Then, primer combination screening involving nine groups identified F3R2 as the most effective primer set, while dilution ratio experiments determined that a 10-fold dilution yielded the best amplification products. Our findings demonstrated that the RPA-LFDA assay had an analytical sensitivity that was capable of detecting as low as 10 target copies per reaction. Furthermore, cross-reactivity tests demonstrated no interference between feline herpesvirus-1 (FHV-1) and feline calicivirus (FCV). To validate our newly developed method against existing techniques in clinical samples from three common sources on the market, we observed superior sensitivity and specificity compared to those of the colloidal gold method (CGM), with a higher positive detection rate using our nucleic acid detection system than CGM. Compared to qPCR as a reference standard, RPA-LFDA detected 39 out of 44 positive samples (including one false positive), whereas CGM detected 26 out of 44 positive samples. Based on the RPA-LFDA, the sensitivity was calculated to be 100%, the specificity was 83.33%, the mistake diagnostic rate was 16.67%, the omission diagnostic rate was 0%, and the overall accuracy reached 97.73%. Moreover, the positive coincidence rate was 97.44%, while the negative coincidence rate reached 100%. The agreement *κ* value was 0.8962. In conclusion, this approach exhibits greater sensitivity than CGM and offers greater convenience and cost-effectiveness than the qPCR methodology, making it a viable option for the clinical detection of FPV.

## 1. Introduction

Feline panleukopenia (FP) is a severe infectious disease that affects both kittens and adult cats [1]. It is caused by a small and minute virus belonging to the *Parvoviridae* family that spreads systemically following oronasal infection [2]. Clinical manifestations of the feline panleukopenia virus (FPV) infection include profound depression, vomiting, diarrhea, a significant reduction in circulating white blood cells (WBCs), and destruction of the intestinal mucosa, leading to enteritis, dehydration, a marked decrease in circulating WBCs, and ultimately mortality. This disease poses significant threats to both cat breeding and wildlife conservation on a global scale [3, 4]. FPV is a nonenveloped single-stranded DNA virus with a genome that contains hairpin structures. Within the genome, the central region encodes two types of proteins: nonstructural proteins (NS1 and NS2) and structural proteins (VP1 and VP2). The NS1 protein plays a pivotal role in viral replication, while the structural VP2 protein has six crucial functions. The VP2 protein and its corresponding coding gene are commonly employed for FPV detection [5, 6, 7].

PCR-based diagnostic methods, including conventional PCR and real-time PCR, have been extensively utilized for the highly sensitive and specific detection of FPV [8, 9, 10]. These procedures typically involve three steps (denaturation, annealing, and extension) performed in multiple cycles. However, these methods are costly, time-consuming, and unsuitable for resource-limited laboratories or field settings. Recently introduced isothermal amplification techniques offer alternative approaches for virus detection. For instance, loop-mediated isothermal amplification (LAMP) enables accurate FPV detection with results comparable to those of real-time PCR but necessitates the use of four or more primers and involves complex primer design [11, 12, 13]. The development of recombinase polymerase amplification (RPA) represents a significant advancement in nucleic acid-based testing due to its rapidity, specificity, and diverse readout options while eliminating the need for comprehensive thermal cycling [14, 15, 16, 17].

The recombinase polymerase amplification–lateral flow dipstick assay (RPA–LFDA) system employs a recombinase and its cofactor to bind with oligonucleotide primers in search of homologous DNA [18, 19]. Subsequently, strand exchange occurs following recognition and binding by the single-strand binding (SSB) protein, which then associates with the parental strand to facilitate amplification using the template strand. DNA polymerase initiates template synthesis from the 3′-terminus of primers to generate new duplex DNA molecules. Through repetitive cycles, specific fragments undergo exponential amplification [20, 21, 22].

Recombinase-based isothermal amplification was conducted within a temperature range of 37−42°C [23]. A detectable amplification signal can be detected within a rapid time frame of 10–20 min via gel electrophoresis, real-time monitoring, or visualization with a lateral flow dipstick (LFD) [24]. Real-time detection incorporates a fluorogenic probe in addition to forward and reverse primers, where the reaction initiates based on the cleavage of the probe at an abasic site (i.e., dSpacer) [25].

In this study, we employed the RPA–LFDA to develop an efficient method for detecting FPV. The rapidity and sensitivity of this method make it highly promising for the early diagnosis of FPV infection in cats.

## 2. Materials and Methods

### 2.1. Source of Primers and Other Materials

The primers were synthesized by Sangon Biotech (Shanghai, China), while the probes were synthesized by General Biol (Anhui, China). The 2 × SanTaq PCR Master Mix (with Blue Dye) was purchased from Sangon Biotech (Shanghai, China). SYBR qPCR mix (lot#F3722) was obtained from NovaBio (Shanghai, China). The RPA kit was obtained from LeSun Bio (Wuxi, China). For LFD scanning and reading, a HIT-91A instrument manufactured by BIOHIT Healthcare Co., Ltd. (Hefei) was utilized. Fluorescent microspheres consisting of highly fluorescent europium (III) nanoparticles (lot#15775) were procured from Bangs Laboratories, Inc. bovine serum albumin (BSA) was acquired from Biosharp Life Science (lot#23212487), rabbit anti-FITC antibody was obtained from BiossTM (Beijing, China), goat anti-rabbit IgG was obtained from Artron (Shandong, China), streptavidin was obtained from Sangon Biotech (Shanghai, China), and MES buffer solution was obtained from BIOFROX. Additionally, the 2 × S6 Universal SYBR qPCR Mix used in this study was purchased from EnzyArtisan (lot# F3722).

### 2.2. Nucleic Acid Extraction

According to the manufacturer's instructions, viral nucleic acids were extracted using the TIANamp Virus DNA/RNA Kit (lot#X0805, Tiangen, China). Reverse transcription was performed to synthesize first-strand cDNA following the manufacturer's instructions using the HyperScript III 1st Strand cDNA Synthesis Kit with gDNA Remover (lot#F3657, Enay Artisan, China). The samples were subsequently stored at −80°C.

### 2.3. Coupling of Biomolecules to Microspheres

First, 1 mL (100 mg/mL) of microspheres was washed twice in 10 mL of activation buffer (10 mM MES, pH 6.0). After all, the pellet was resuspended in 10 mL of activation buffer to ensure thorough suspension of the microspheres. While continuously mixing, 100 mg of water-soluble carbodiimide (WSC, 1-ethyl-3- (3-dimethylaminopropyl) carbodiimide hydrochloride) was added. The reaction was carried out at 25°C for 15 min with constant stirring. Subsequently, the solution was washed twice using coupling buffer (10 mM phosphate buffered saline, pH = 7.4) and then resuspended in an equal volume of the same buffer solution (5 mL). Proteins (in excess by a factor of 10 compared to the calculated monolayer) were solubilized in another solution containing 5 mL of coupling buffer. The microsphere suspension was mixed with the protein solution and stirred continuously at 25°C for 3 hr. The mixture underwent washing and was subsequently resuspended in a quenching solution comprising ethanolamine (30 mM) and BSA at a concentration of 1% w/v. The resuspension was stirred gently for 30 min. Finally, the sample was washed and resuspended in a storage buffer containing phosphate buffer with a concentration of 0.1% w/v BSA. It can be safely stored at temperatures as low as 4°C until further use.

### 2.4. RPA–LFDA Test Method

**Figure 1:**
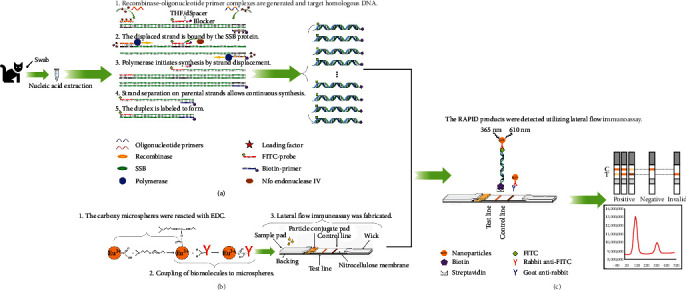
An overview of the experimental approach employed by the RPA–LFDA system for the detection of feline panleukopenia virus (FPV). (a) Recombinase-based isothermal amplification was employed to amplify double-stranded DNA products labeled with fluorescein isothiocyanate (FITC) and biotin using DNA as the template. (b) Biomolecules were conjugated to microspheres via the coupling of biomolecules and subsequently applied onto the test strip, while the immobilization of streptavidin and goat anti-rabbit antibody occurred at the T-line and C-line positions simultaneously. (c) The RAPID product was applied to the strip, and the fluorescence intensity of the T and C-lines was quantified using a HIT-91A instrument.

The RAPID reactions, as shown in Figure 1, were conducted using LeSun Bio (Wuxi, China). All reaction mixtures were prepared with a final volume of 50 *μ*L, comprising 2.1 *μ*L of forward primer (10 *μ*M), 2.1 *μ*L of reverse primer (10 *μ*M), 0.6 *μ*L of probe (10 *μ*M), 3.0 *μ*L of magnesium acetate (280 mM), lyophilized enzyme powder, 15.2 *μ*L of ddH_2_O, and 25 *μ*L of buffer, along with either genomic DNA or plasmid at a concentration of 2 *μ*L each. To prepare the RPA-Mix solution included in the kit, each tube was supplemented with 25 *μ*L of buffer and mixed well with an additional 15.2 *μ*L of ddH_2_O to completely dissolve the white powder before subsequent experiments were performed. The procedure for the RPA reactions involved incubating the tubes at an optimal reaction temperature for 4 min, followed by removal from the thermostat for manual flicking and brief centrifugation before the tubes were allowed to react for an additional 15 min.

#### 2.4.1. Analysis of Optimal Primer Combinations

**Figure 2:**
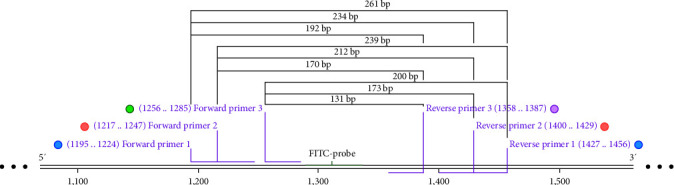
Primer and probe design for the RPA–LFDA assay system. A set of three forward and three reverse primers was designed to target the FPV-VP2 gene, resulting in the amplification of nine nucleic acid fragments: F1R1 (261 bp), F1R2 (234 bp), F1R3 (192 bp), F2R1 (239 bp), F2R2 (212 bp), F2R3 (170 bp), F3R1 (200 bp), F3R2 (173 bp), and F3R3 (131 bp).

To assess primer validity, the recombinant plasmid pET-FPV-VP2 was utilized as a template, and the designed forward and reverse primers were paired for PCR at an annealing temperature of 50°C. The RPA method was employed to screen the primers using a fluorescent-type nucleic acid amplification kit (NovaBio, China). The procedure involved combining 25 *μ*L of deliquescent agent, 2.1 *μ*L of each FPV forward and reverse primer (10 *μ*mol/L), 0.6 *μ*L of FPV-Probe, 2 *μ*L of template, 3 *μ*L of activator, and ddH_2_O to make up a total volume of 50 *μ*L. Subsequently, the mixture was promptly loaded into a real-time fluorescence quantitative PCR instrument, where SYBR fluorescence information was collected every 5 s at a temperature of 39°C. Triplicate tests were conducted to obtain average cycle threshold (Ct) values for the primers to identify the most efficient primer pairs (Figure 2, Table 1).

#### 2.4.2. Dilution Analysis of the RPA Products

The highly viscous amplification products needed to be diluted to test the purpose of the nucleic acid amplification products. At the end of the reaction, the RPA amplification products were diluted 1.4-fold, 5-fold, 10-fold, 20-fold, 30-fold, 40-fold, 50-fold, 60-fold, 70-fold, 80-fold, 90-fold, and 100-fold with PBST buffer, and the diluted products were examined with LFD. The dilution method is as shown in Table 2.

#### 2.4.3. RPA Temperature Analysis

The following steps were performed to determine the optimal reaction temperature: Eight temperatures (25, 28, 30, 33, 36, 39, 42, and 45°C) were used for the RPA reaction, which was controlled by a PCR instrument. At the end of the reaction, the RPA product was diluted 10-fold using PBST and detected by a lateral chromatography dipstick, and the T-line and C-line fluorescence intensities were read by a HIT-91A instrument.

### 2.5. Sensitivity, Specificity, and Limit of Blank (LoB) Analysis

The copy number of FPV was calculated by the following formula: amount (copies/*µ*L) = 6 × 10^23^ (copies/mol) × concentration (g/*µ*L)/MW (g/mol). For analysis of sensitivity, the recombinant plasmids were diluted from 10^0^ to 10^6^ copies/*µ*L and subsequently tested by the RPA method. DNA or cDNA from FPV, FHV-1, and FCV was extracted from CRFK cell culture supernatants and used for RPA specificity analysis to eliminate cross-reactions between the RPA method and other feline viruses or genomes. The RPA assay system was tested with 20 PBST buffer (10 mM PBS, 0.05% Tween 20, pH 7.4) samples, the results were recorded on a HIT-91A instrument, and the data were collated and analyzed. The following formula was used to calculate LoB: Mean + 2 × STDEV.

### 2.6. Clinical Sample Analysis

Forty-four nasopharyngeal swabs from cats were collected from pet hospitals in Hefei, Anhui to evaluate the performance of the RPA–LFDA method in the detection of FPV in clinical samples, and the results were compared with real-time quantitative PCR (qRT-PCR) and the colloidal gold method (CGM) strip test. All the samples were stored in a −80°C freezer. The formula for calculating the relevant detection indicators is presented in Table 3, where a, b, c, and d are the number of samples counted. Sensitivity = a/(a + c)100%; specificity = d/(b + d)100%; *α*, mistake diagnostic rate = b/(b + d)100%; *β*, omission diagnostic rate = c/(a + c)100%; accuracy = (a + d)/(a + b + c + d)100%; positive coincidence rate = a/(a + b)100%; negative coincidence rate = d/(c + d)100%; and agreement *κ* value = 2(ad−bc)/((a + b) (b + d) (a + c) (c + d)).

## 3. Results

### 3.1. Analysis of Optimal Primer Combinations

**Figure 3:**
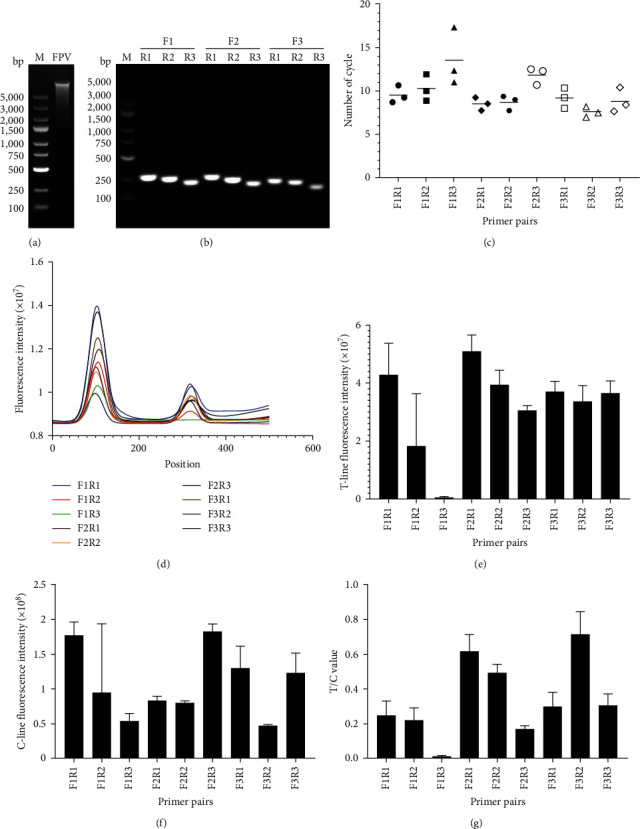
Results of FPV nucleic acid extraction and primer screening. (a) Results of FPV nucleic acid extraction by the TIANamp Virus DNA Kit. (b) Different primer combinations for PCR amplification. The amplified fragments were visualized by agarose gel electrophoresis. (c) Fluorescence intensity was monitored during amplification using a qRT-PCR instrument. SYBR Green I fluorescent dye was added to the RAPID amplification reaction mixture, and the resulting Ct value was subjected to statistical analysis. F3R2 had the smallest Ct value. (d) RPA amplification results under different primer combinations were tested, and the test strips were fully scanned by a HIT-91A instrument. The fluorescence intensities of the T-lines (e), C-lines (f) and T/C (g) were recorded and analyzed based on different primer combinations.

Three pairs of a total of nine primer combinations were designed for validation, as shown in Table 1. The optimal primers for the RPA–LFDA were screened by a series of PCR tests. First, the FPV virus nucleic acid genome was extracted, and the results were subjected to agarose gel electrophoresis (Figure 3(a)). Each primer pair demonstrated efficient PCR amplification (Figure 3(b)). Second, SYBR Green I fluorescent dye was added to the RPA nucleic acid amplification system to monitor the amplification results (Figure 3(c)). To visualize the performance of the primers, the Ct value at which the fluorescence intensity reached the positive threshold was plotted on the *Y*-axis, while the serial number of each primer pair was plotted on the *X*-axis. Under these conditions, the lower the Ct and SD values were, the better the amplification efficiency and stability. Among all primer combinations, F3R2 had the smallest Ct value. This primer combination had relatively high amplification efficiency (Figure 3(c)). Third, each primer pair was tested using the RPA method. Full scans of the test strips, including the T-line and C-line results, were recorded on a HIT-91A instrument, and the data were collated and analyzed (Figures 3(d), 3(e), 3(f), and 3(g)). Finally, F3R2 had the lowest and most stable Ct values. In summary, the primer pair F3R2 was chosen as the optimal primer pair for the RPA method.

### 3.2. Dilution Analysis of the RPA Products

**Figure 4:**
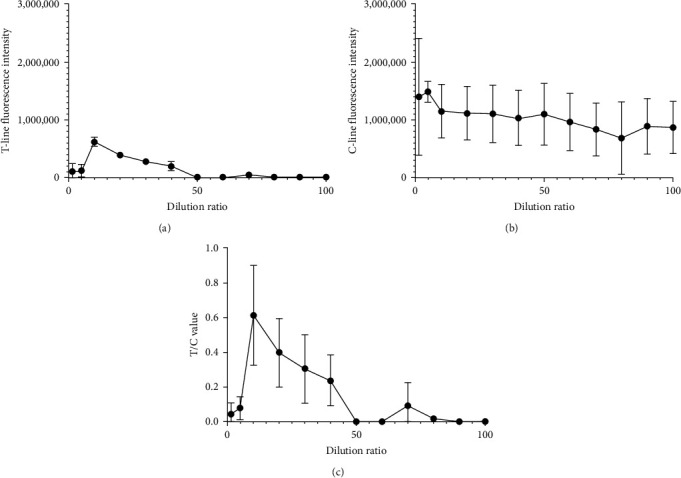
Dilution method for RPA product dilution. (a) Detection of the fluorescence intensity of the T-line was performed at various dilution ratios to determine the experimental outcomes. (b) Fluorescence intensity detection of the C-line was performed under various dilution ratios to obtain the experimental outcomes. (c) Fluorescence intensity detection of the T/C value was performed under various dilution ratios to obtain the experimental outcomes.

RPA nucleic acid amplification products were diluted as described in Table 2. The diluted sample was tested by a strip. The assay results were recorded by a HIT-91A instrument, as shown in Figure 4. The RPA nucleic acid amplification products had relatively low T/C values at dilutions less than 10 or greater than 40-fold. However, a 10-fold dilution ratio had the highest T/C value. Therefore, the optimal dilution of the RPA nucleic acid amplification products in this detection system is 10-fold.

### 3.3. RPA Temperature Analysis

**Figure 5:**
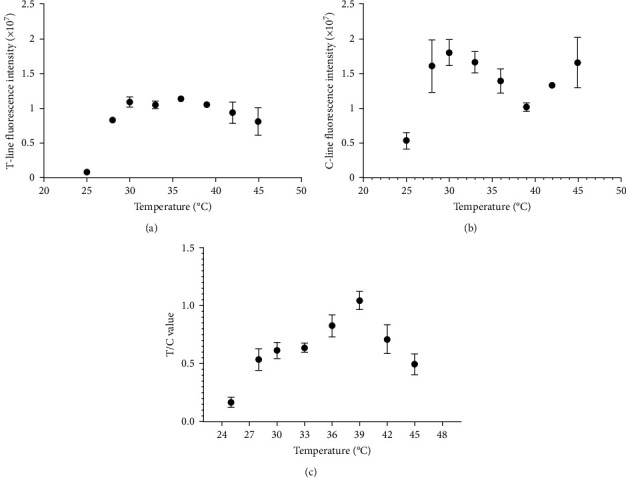
Optimal temperature screening for the RPA detection system. (a) Results of T-line fluorescence intensity under varying temperature conditions. (b) Results of C-line fluorescence intensity under varying temperature conditions. (c) Results of the T/C values under different temperature conditions.

The detection system was set at different reaction temperatures. Within a certain temperature range, the amplification efficiency of nucleic acids increased with increasing temperature (Figure 5). The test results were recorded by a HIT-91A instrument, ranging from 25 to 39°C, and the amplification efficiency tended to increase; in the 39–45°C range, the amplification efficiency decreased (Figure 5(c)). The findings demonstrated that the optimal amplification temperature for the detection system was 39°C.

### 3.4. Sensitivity, Specificity, and LoB Analysis

**Figure 6:**
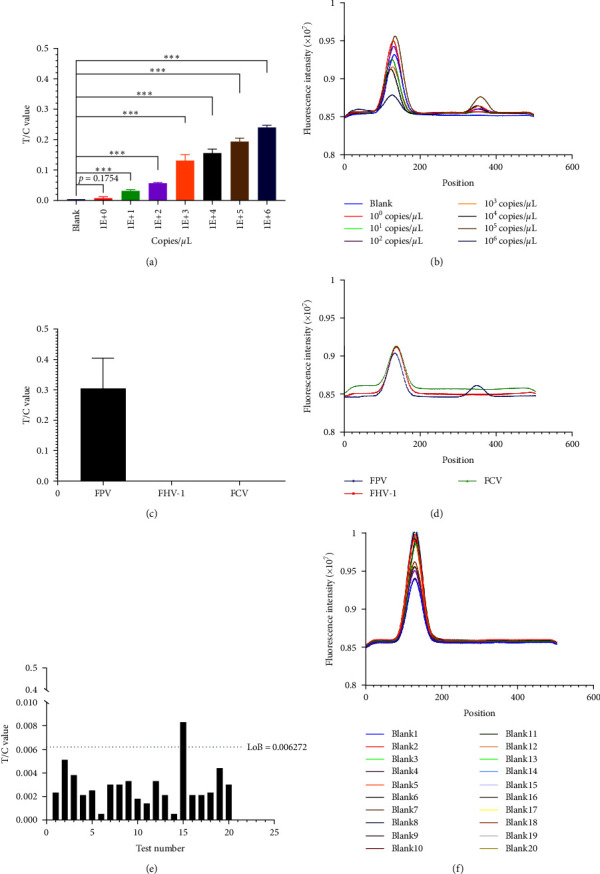
Assessment of the analytical performance of the RPA–LFDA detection system. (a) Results of the T/C value for RPA–LFDA detection utilizing plasmids with varying copy numbers as templates ( ^*∗*^*p* < 0.05). (b) The test strips were scanned after completion of the sensitivity testing process by a HIT-91A instrument. (c) The specificity of the RPA–LFDA system was assessed using FPV, FHV-1, and FCV nucleic acids. (d) The test strips were scanned after completion of the specificity testing process by a HIT-91A instrument. (e) To assess the detection limit of the RPA-LFDA detection system, a set of 20 blank samples was used as templates for isothermal amplification, followed by subsequent detection of the amplification products using the test strip. (f) The test strips were scanned after completion of the LoB testing process by a HIT-91A instrument. The symbol “ ^*∗∗∗*^” signifies the extremely significantly different *p* value = 0.0004.

For sensitivity, copy numbers ranging from 10^6^ to 10^0^ of the plasmids were amplified by the established RPA procedure. First, the initial template concentration was determined using a calculation formula. Subsequently, the recombinant plasmid with a predetermined copy number was used as an amplification template for the RPA nucleic acid amplification system. Amplification was conducted at 39°C utilizing the primer F3R2. The resulting amplification products were diluted 10-fold and subsequently detected using strip analysis and the HIT-91A instrument. Compared with those in the control group, the number of genes in the 101-gene group significantly differed (Figures 6(a) and 6(b)). The results showed that the sensitivity of detection was 10^1^ copies. For specificity, only FPV was amplified by the established RPA method. Notably, FHV-1 and FCV did not cross-react (Figures 6(c) and 6(d)), which indicates that the established FPV-RPA method has excellent specificity. The LoB analysis for the RPA method was determined for accurate diagnosis of the template. In this experiment, the LoB of the RPA–LFDA assay was determined using 20 blank samples. These 20 samples were added to the RPA–LFDA as templates, and the test results were recorded by a HIT-91A instrument. According to the LoB = M + 2SD calculation, the result was 0.006272 (Figures 6(e) and 6(f)).

### 3.5. Clinical Sample Analysis

**Figure 7:**
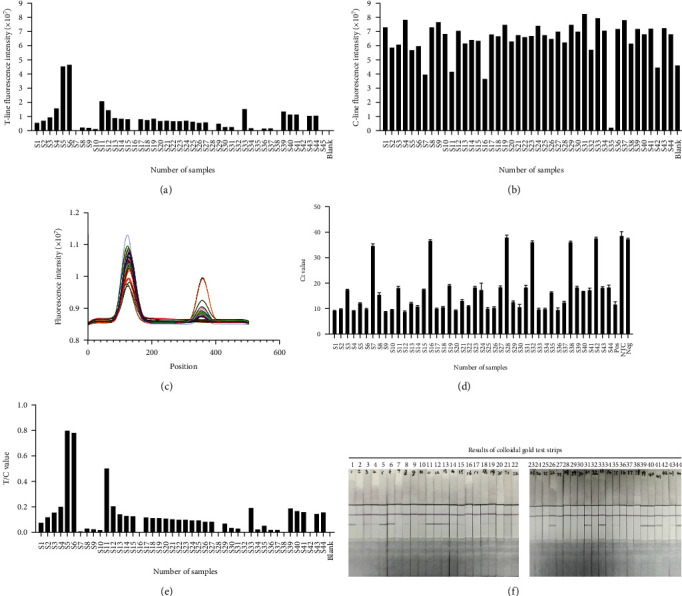
The three assays were utilized to examine a total of 44 clinical samples. (a) The T values of the 44 specimens were ascertained through the application of the RPA–LFDA. (b) The C values of the 44 specimens were ascertained through the application of the RPA–LFDA. (c) The T/C values of the 44 specimens were ascertained through the application of the RPA–LFDA. (d) The test strip was subjected to scanning via the RPA–LFDA technique facilitated by the HIT-91A instrument. (e) Ct values were recorded using qRT-PCR of 44 samples for testing. (f) The results of 44 samples tested by the CGM assay were recorded.

Forty-four oral swabs from cats were collected for testing using the RPA–LFDA method (Figures 7(a), 7(b), 7(c), and 7(d)). In total, 86.64% (39/44) of the samples were positive. All of the positive and negative samples were verified by qRT-PCR, which detected 38 positive and 6 negative samples (Figure 7(e)). At the same time, all the samples were tested using colloidal gold strips, and the results were statistically compared; 26 positive and 18 negative results were obtained (Figure 7(f), and Table 4). Compared with CGM, the RPA–LFDA method exhibits superior detection sensitivity, enhanced detection accuracy, and an improved negative coincidence rate. Furthermore, the results obtained from this approach align with those derived from qRT-PCR analysis. The statistical comparison of the detection results of 44 samples by qRT-PCR, RPA–LFDA, and CGM are shown below (Table 5). The results showed that the sensitivity of the RPA–LFDA was greater than that of the CGM (Table 5; 38,100% for the RPA–LFDA and 26,68.42% for the CGM). Both the RPA–LFDA and CGM had good specificity (RPA–LFDA, 83.33% and CGM, 100%). In contrast, CGM had a higher omission diagnostic rate. In addition, the RPA–LFDA showed better accuracy. The above results indicate that the RPA–LFDA may be a more accurate detection method than CGM.

**Figure 8:**
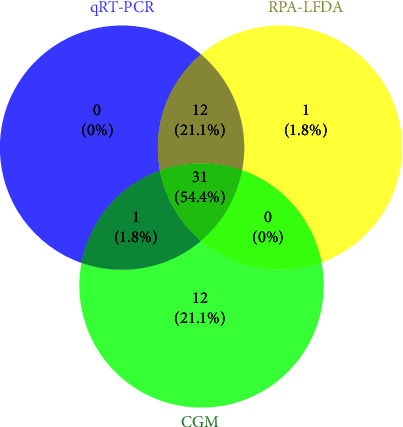
Venn diagram of the number of FPVs identified via qRT-PCR, RPA–LFDA, and CGM.

We detected positive and negative results for 44 samples (Figure 8), 43 of which were consistent with the qRT-PCR and RPA–LFDA results. Simultaneously, we obtained only 32 congruent outcomes shared by qRT-PCR and CGM. Upon examination of the graphical data, it is evident that a solitary instance of RPA–LFDA results exhibited qRT-PCR outcomes, in contrast to 21 instances of CGM results, which demonstrated noncongruence with the PCR benchmarks. By comparative analysis with the RPA–LFDA and CGM methodologies, it was discerned that only 31 detection outcomes were in alignment with the expected consistency.

## 4. Discussion

Currently, there is a continuous upward trend in the proportion of pet cats within households [26]. FPV is one of the most severe viral infections and poses a significant threat to feline health [27, 28]. Notably, households with multiple cats and those with kittens exhibit high morbidity rates. Despite the availability of a vaccine, it appears that cats possess lower immunity against this virus than initially anticipated. Consequently, the development of a rapid and simultaneous detection method for FPV is essential for both prevention and control strategies targeting these two viral pathogens [29, 30, 31].

The RPA–LFDA technique enables the amplification of target gene fragments at a constant temperature, eliminating the need for thermal cycling. In comparison to traditional PCR and other isothermal nucleic acid amplification methods, RPA–LFDA offers significant advantages in terms of rapid expansion time. Furthermore, multiple inspection equipment is needed because the results can be displayed within 5 min using agarose gel electrophoresis or LFD [32, 33]. Due to these remarkable characteristics, this technique has been successfully applied in detecting various pathogens, including viruses, bacteria, fungi, mycoplasma, and Plasmodium falciparum.

The RPA–LFDA platform integrates diverse technologies to address a wide range of detection needs. For instance, reverse transcription RPA–LFDA (RT-RPA) employs modified RNA reverse transcriptase for efficient RNA conversion. Moreover, DNA recombinases streamline and enhance assay reliability by initiating DNA synthesis using cDNA as a template. When coupled with LFD technology or the CRISPR-Cas12a system, RPA enables rapid pathogen identification and generates visible LED blue light signals. Additionally, when combined with Exo probes, RPA allows real-time detection of various pathogens with exceptional specificity and sensitivity while offering superior speed, portability, and accessibility compared to qRT-PCR.

Finally, the point-of-care (POC) application of RPA testing confers significant advantages. However, it is important to acknowledge certain limitations. First, prior to conducting nucleic acid testing, specific requirements such as DNA/RNA extraction and sample reverse transcription must be met. Second, due to its rapid response speed, the fluorescence in the RPA-LFDA reached a plateau within a short period. Therefore, it is crucial to promptly measure changes in fluorescence values after the addition of all reagents.

## 5. Conclusion

The results demonstrated the successful identification of a rapid nucleic acid detection system utilizing F3R2 primer combinations and amplification at 39°C. Following a 10-fold dilution, the target double-stranded DNA content was effectively detected using LFD. The rapid RPA–LFDA nucleic acid detection system exhibited a sensitivity of 10^1^ copies/*μ*L without any cross-reactivity to FHV-1 or FCV, thereby demonstrating excellent specificity. Evaluation with 44 clinical samples showed robust detection performance compared to qRT-PCR as a reference, with a sensitivity of 100%, specificity of 83.33%, and accuracy of 97.73%. The RPA–LFDA nucleic acid detection protocol established in this study can significantly enhance the efficiency of panleukopenia nucleic acid detection in cats.

## Figures and Tables

**Table 1:** Primers and probe for the RAPID method.

Primer^a^	Primer sequences (5′-3′)^b^
FPV-F1	ACATATATAGCACATCAAGATACAGGAAGA
FPV-F2	CAGGAAGATATCCAGAAGGAGATTGGATTCA
FPV-F3	ACTTTAACCTTCCTGTAACAAATGATAATG
FPV-R1	5′Biotin-CATTTACATGAAGTCTTGGTTTTAAGTCAG
FPV-R2	5′Biotin-CAGTATCAAATTCTTTATCCCAAATTTGAC
FPV-R3	5′Biotin-CTGGTGGTACATTATTTAATGCAGTTAAAG
FPV-Probe	5′FITC-GCTACCAACAGATCCAATTGGAGGTAAAAC/dSpacer/GGAATTAACTATACTA-C3 Spacer

^a^F, forward primer and R, reverse primer. ^b^FITC, fluorescein isothiocyanate and dSpacer, tetrahydrofuran.

**Table 2:** Dilution method for RPA product dilution.

Reagent name	Dilution ratio											
	1.4	5	10	20	30	40	50	60	70	80	90	100
RPA products (*μ*L)	50.00	14.00	7.00	3.50	2.33	1.75	1.40	1.17	1.00	0.88	0.77	0.70
PBST (*μ*L)	20.00	56.00	63.00	66.50	67.67	68.25	68.60	68.83	69.00	69.12	69.23	69.30

**Table 3:** The *χ^2^* test was conducted on a four-grid table of data.

Method	Sample	qRT-PCR		
		P	N	T
RAPID/CGM	P	a	b	a + b
	N	c	d	c + d
	T	a + c	b + d	a + b + c + d

P, positive; N, negative; T, total; a, both qRT-PCR and RAPID or colloidal gold method (CGM) positive; b, qRT-PCR negative and RAPID or CGM positive; c, qRT-PCR positive and RAPID or CGM negative; and d, both qRT-PCR and RAPID or CGM negative.

**Table 4:** The results of 44 clinical samples were analyzed using three detection methods, encompassing both positive and negative outcomes.

Sample ID	Sample type	qPCR		RPA–LFDA		CGM	
		Ct value	Result	T/C value	Result	T color ^*∗*^	Result
S1	Oral swab	9.22	+	0.0752	+	1	+
S2	Oral swab	9.83	+	0.1188	+	1	+
S3	Oral swab	17.42	+	0.1543	+	1	+
S4	Oral swab	9.10	+	0.2014	+	1	+
S5	Oral swab	12.06	+	0.7962	+	1	+
S6	Oral swab	9.87	+	0.7793	+	1	+
S7	Oral swab	34.63	−	0.0068	+	0	−
S8	Oral swab	15.44	+	0.0296	+	1	+
S9	Oral swab	8.82	+	0.0248	+	0	−
S10	Oral swab	9.61	+	0.0173	+	1	+
S11	Oral swab	18.05	+	0.4992	+	1	+
S12	Oral swab	8.83	+	0.2044	+	1	+
S13	Oral swab	12.14	+	0.1431	+	1	+
S14	Oral swab	10.84	+	0.1311	+	1	+
S15	Oral swab	17.49	+	0.1261	+	1	+
S16	Oral swab	36.51	−	0.0003	−	0	−
S17	Oral swab	9.98	+	0.1168	+	0	−
S18	Oral swab	10.62	+	0.1132	+	1	+
S19	Oral swab	19.08	+	0.1129	+	1	+
S20	Oral swab	9.31	+	0.1081	+	0	−
S21	Oral swab	13.01	+	0.1041	+	1	+
S22	Oral swab	10.94	+	0.0989	+	1	+
S23	Oral swab	18.26	+	0.0977	+	0	−
S24	Oral swab	17.32	+	0.0945	+	0	−
S25	Oral swab	10.01	+	0.0943	+	0	−
S26	Oral swab	10.37	+	0.0843	+	1	+
S27	Oral swab	18.35	+	0.0833	+	0	−
S28	Oral swab	37.89	−	0.0001	−	0	−
S29	Oral swab	12.58	+	0.0671	+	0	−
S30	Oral swab	10.63	+	0.0358	+	0	−
S31	Oral swab	18.28	+	0.0302	+	1	+
S32	Oral swab	36.03	−	0.0001	−	0	−
S33	Oral swab	9.64	+	0.1918	+	1	+
S34	Oral swab	9.84	+	0.0241	+	0	−
S35	Oral swab	16.38	+	0.0219	+	1	+
S36	Oral swab	9.52	+	0.0204	+	0	−
S37	Oral swab	12.53	+	0.0197	+	0	−
S38	Oral swab	36.12	−	0.0001	−	0	−
S39	Oral swab	18.38	+	0.1884	+	1	+
S40	Oral swab	16.75	+	0.1672	+	1	+
S41	Oral swab	17.32	+	0.1586	+	1	+
S42	Oral swab	37.54	−	0.0001	−	0	−
S43	Oral swab	18.26	+	0.1436	+	1	+
S44	Oral swab	18.28	+	0.1562	+	1	+
Total	—	—	P (38), N (6)	—	P (39), N (5)	—	P (26), N (18)

T color ^*∗*^, 1 indicates that the T line can be found, representing a positive result and 0 indicates that the T line cannot be found, representing a negative result.

**Table 5:** Statistics on detection indicators in clinical samples.

Detection method	qRT-PCR			Sensi (%)	Speci (%)	*α* (%)	*β* (%)	Acc (%)	Pcr (%)	Ncr (%)	K
	P	N	T								
RPA–LFDA											
P	38	1	39	100.00	83.33	16.67	0.00	97.73	97.44	100.00	0.8962
N	0	5	5								
T	38	6	44								
CGM											
P	26	0	26	68.42	100.00	0.00	31.57	72.73	100.00	33.33	0.3714
N	12	6	18								
T	38	6	44								

P, positive; N, negative; T, total; Sensi, sensitivity; Speci, specificity; *α*, mistake diagnostic rate; *β*, omission diagnostic rate; Acc, accuracy; Pcr, positive coincidence rate; Ncr, negative coincidence rate; and K, agreement *κ* value.

## Data Availability

The data presented in this study are available upon request from the corresponding author.

## Additional Points

*Simple Summary*. Feline panleukopenia virus (FPV) is a prevalent pathogenic virus in cats. Currently, there is an urgent demand for a rapid, highly sensitive, and convenient detection method. In response to this need, we developed a novel recombinase polymerase amplification–lateral flow dipstick assay (RPA–LFDA). Current detection methods, such as the colloidal gold method (CGM) test strips and polymerase chain reaction (PCR), are limited in terms of convenience and sensitivity. RPA–LFDA was developed by fluorescein isothiocyanate (FITC)-modified probes and biotin-modified reverse primers. Ultimately, the integration of europium(III) fluorescent nanosphere test strips enabled rapid quantitative detection of FPV. This RPA–LFDA demonstrated excellent performance with a detection limit of 10^1^ copies for FPV without any cross-reactivity with feline calicivirus (FCV) or feline herpes virus-1 (FHV-1). To assess its clinical utility, we collected and tested 44 nasopharyngeal swabs from cats. The positive rate and accuracy rate correlated well with the results obtained using quantitative real-time PCR analysis. Therefore, this RAPID method represents a novel and efficient clinical diagnostic tool for the accurate detection of FPV. *Highlights*. RPA–LFDA exhibited high specificity (83.33%) and sensitivity (100%), achieving a detection limit of 10^1^ copies within a short duration of 15 min. RPA enables nucleic acid amplification to be completed at 39°C, with products achieved within a mere 2 min utilizing a europium(III) fluorescent nanosphere test strip. RPA–LFDA has significant potential for further development with the goal of achieving rapid nucleic acid detection with portable devices in field settings.

## Supplementary Materials

Supplementary 1 Figure 1: assessment of the analytical performance of the RPA-LFDA detection system.

Supplementary 2 Figure 2: dilution method for RPA product dilution.

Supplementary 3 Figure 3: optimal temperature screening for RPA detection system.

Supplementary 4 Figure 4: original images for gels.

Supplementary 5 Figure 5: results of FPV nucleic acid extraction and primer screening.

Supplementary 6 Figure 6: the three assays were utilized to examine a total of 44 clinical samples.

## References

[1] Roozitalab A., Elsakhawy O. K., Abouelkhair M. A., Matthijnssens J. M. (2023). Complete coding sequence of two feline panleukopenia virus strains isolated from domestic cats (*Felis catus*) in Tennessee, USA. *Microbiology Resource Announcements*.

[2] Pan S., Jiao R., Xu X. (2023). Molecular characterization and genetic diversity of parvoviruses prevalent in cats in Central and Eastern China from 2018 to 2022. *Frontiers in Veterinary Science*.

[3] Zhao S., Hu H., Lan J. (2023). Characterization of a fatal feline panleukopenia virus derived from giant panda with broad cell tropism and zoonotic potential. *Frontiers in Immunology*.

[4] Kabir A., Habib T., Chouhan C. S. (2023). Epidemiology and molecular characterization of feline panleukopenia virus from suspected domestic cats in selected Bangladesh regions. *PLOS ONE*.

[5] Li S., Chen X., Hao Y. (2022). Characterization of the VP2 and NS1 genes from canine parvovirus type 2 (CPV-2) and feline panleukopenia virus (FPV) in Northern China. *Frontiers in Veterinary Science*.

[6] Shao R., Ye C., Zhang Y. (2021). Novel parvovirus in cats, China. *Virus Research*.

[7] Pfankuche V. M., Jo W. K., van der Vries E. (2018). Neuronal vacuolization in feline panleukopenia virus infection. *Veterinary Pathology*.

[8] Xue H., Liang Y., Gao X. (2023). Development and application of nanoPCR method for detection of feline panleukopenia virus. *Veterinary Sciences*.

[9] Cao N., Tang Z., Zhang X. (2022). Development and application of a triplex taqman quantitative real-time PCR assay for simultaneous detection of feline calicivirus, feline parvovirus, and feline herpesvirus 1. *Frontiers in Veterinary Science*.

[10] Wang Y., Pan Y., Wu J. (2021). Simultaneous detection of feline parvovirus and feline bocavirus using SYBR Green I-based duplex real-time polymerase chain reaction. *3 Biotech*.

[11] Zhang Q., Niu J., Yi S. (2019). Development and application of a multiplex PCR method for the simultaneous detection and differentiation of feline panleukopenia virus, feline bocavirus, and feline astrovirus. *Archives of Virology*.

[12] Abd-Eldaim M., Beall M. J., Kennedy M. A. (2009). Detection of feline panleukopenia virus using a commercial ELISA for canine parvovirus. *Veterinary Therapeutics: Research in Applied Veterinary Medicine*.

[13] Raheena K. P., Priya P. M., Mani B. K., Mini M., Pillai U. N. (2017). Comparison of different diagnostic test to detect feline panleukopenia virus among cats in Kerala, India. *Indian Journal of Animal Research*.

[14] Fan X., Li L., Zhao Y. (2020). Clinical validation of two recombinase-based isothermal amplification assays (RPA/RAA) for the rapid detection of african swine fever virus. *Frontiers in Microbiology*.

[15] Chen B., Zhang H., Wang H., Li S., Zhou P. (2023). Development and application of a dual ERA method for the detection of feline calicivirus and feline herpesvirus type I. *Virology Journal*.

[16] Yang X., Zhao P., Dong Y. (2021). An isothermal recombinase polymerase amplification and lateral flow strip combined method for rapid on-site detection of *Vibrio vulnificus* in raw seafood. *Food Microbiology*.

[17] Liu H., Wang J., Hu X., Tang X., Zhang C. (2022). A rapid and high-throughput *Helicobacter pylori* RPA-CRISPR/Cas12a-based nucleic acid detection system. *Clinica Chimica Acta; International Journal of Clinical Chemistry*.

[18] Li J. S., Hao Y. Z., Hou M. L. (2022). Development of a recombinase-aided amplification combined with lateral flow dipstick assay for the rapid detection of the African swine fever virus. *Biomedical and Environmental Sciences*.

[19] Stephanie E. O. B. M. (2021). A review of reaction enhancement strategies for isothermal nucleic acid amplification reactions. *Sensors and Actuators Reports*.

[20] Wang Z.-H., Wang X.-J., Hou S.-H. (2019). Development of a recombinase polymerase amplification assay with lateral flow dipstick for rapid detection of feline parvovirus. *Journal of Virological Methods*.

[21] Piepenburg O., Williams C. H., Stemple D. L., Armes N. A. (2006). DNA detection using recombination proteins. *PLOS Biology*.

[22] Ghosh D. K., Kokane S. B., Kokane A. D. (2018). Development of a recombinase polymerase based isothermal amplification combined with lateral flow assay (HLB-RPA-LFA) for rapid detection of “*Candidatus Liberibacter asiaticus"*. *PLOS ONE*.

[23] Yukhet P., Buddhachat K., Vilaivan T., Suparpprom C. (2021). Isothermal detection of canine blood parasite (*Ehrlichia canis*) utilizing recombinase polymerase amplification coupled with graphene oxide quenching-based pyrrolidinyl peptide nucleic acid. *Bioconjugate Chemistry*.

[24] Ceruti A., Kobialka R. M., Ssekitoleko J. (2021). Rapid extraction and detection of african swine fever virus DNA based on isothermal recombinase polymerase amplification assay. *Viruses*.

[25] Van Vuuren M., Steinel A., Goosen T. (2000). Feline panleukopenia virus revisited: molecular characteristics and pathological lesions associated with three recent isolates. *Journal of the South African Veterinary Association*.

[26] Csiza C. K., De Lahunta A., Scott F. W., Gillespie J. H. (1971). Pathogenesis of feline panleukopenia virus in susceptible newborn kittens II. Pathology and immunofluorescence. *Infection and Immunity*.

[27] Stuetzer B., Hartmann K. (2014). Feline parvovirus infection and associated diseases. *The Veterinary Journal*.

[28] Pacini M. I., Forzan M., Franzo G. (2023). Feline parvovirus lethal outbreak in a group of adult cohabiting domestic cats. *Pathogens*.

[29] Jacobson L. S., Janke K. J., Giacinti J., Weese J. S. (2021). Diagnostic testing for feline panleukopenia in a shelter setting: a prospective, observational study. *Journal of Feline Medicine and Surgery*.

[30] Kumar S., Kumar A., Venkatesan G. (2018). Isothermal nucleic acid amplification system: an update on methods and applications. *Journal of Genetics and Genomes*.

[31] Garigliany M., Gilliaux G., Jolly S. (2016). Feline panleukopenia virus in cerebral neurons of young and adult cats. *BMC Veterinary Research*.

[32] Burmeister W. P., Buisson M., Estrozi L. F. (2015). Structure determination of feline calicivirus virus-like particles in the context of a pseudo-octahedral arrangement. *PLOS ONE*.

[33] Shelite T. R., Uscanga-Palomeque A. C., Castellanos-Gonzalez A., Melby P. C., Travi B. L. (2021). Isothermal recombinase polymerase amplification-lateral flow detection of SARS-CoV-2, the etiological agent of COVID-19. *Journal of Virological Methods*.

