# Supplementary figures and images for: Development and Application of an RPA-Based Rapid Point-of-Care Testing (POCT) Method for the Detection of Feline Panleukopenia Virus
Source: Transbound Emerg Dis. 2024 Aug 24;2024:3680778. doi: 10.1155/2024/3680778 (PMC12016765; doi:10.1155/2024/3680778)

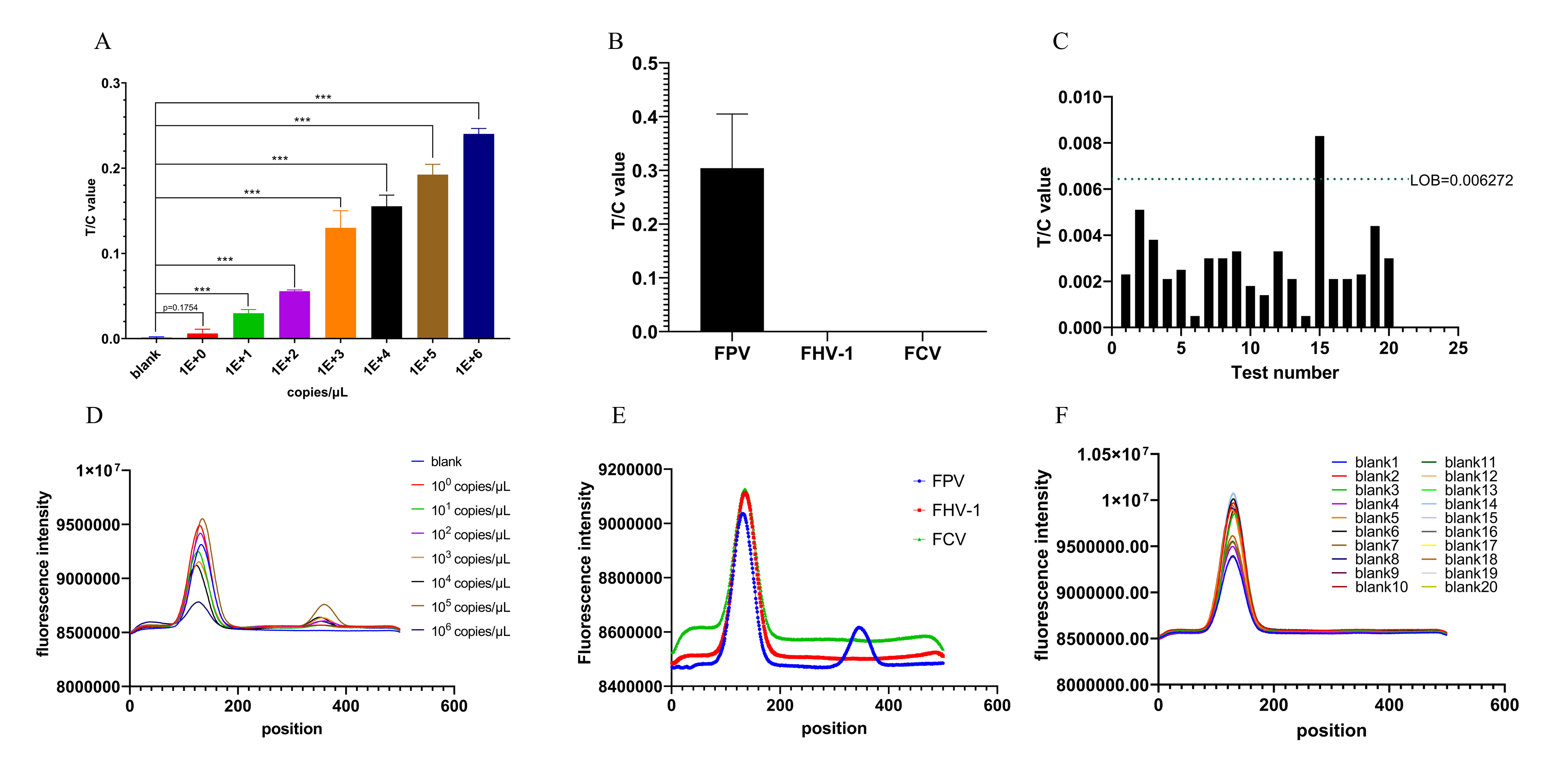

Supplement: Supplementary 1 — Figure 1: assessment of the analytical performance of the RPA-LFDA detection system. [file 3680778.f1.png]

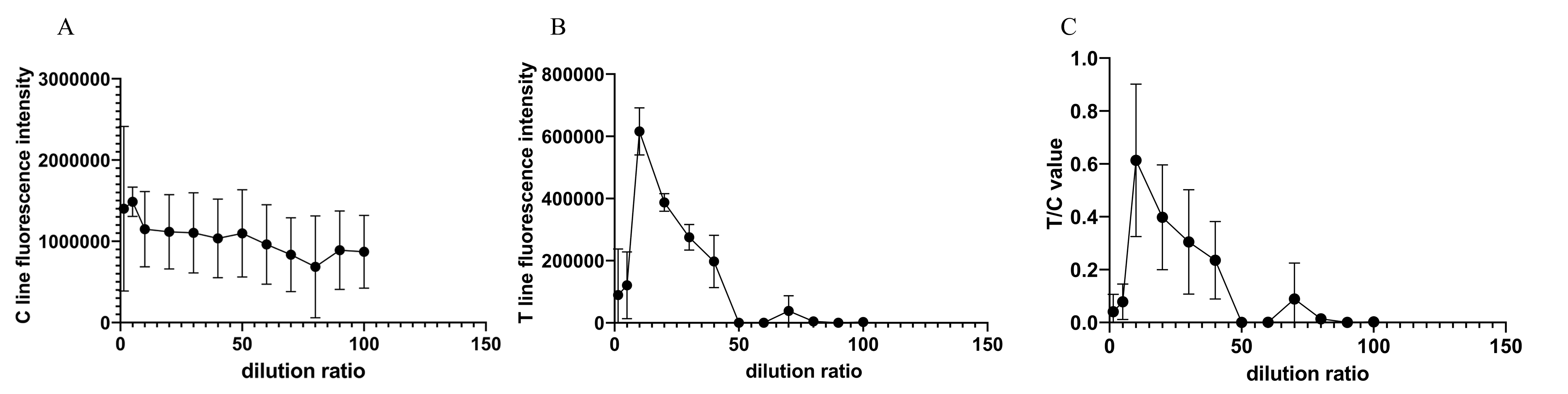

Supplement: Supplementary 2 — Figure 2: dilution method for RPA product dilution. [file 3680778.f2.jpg]

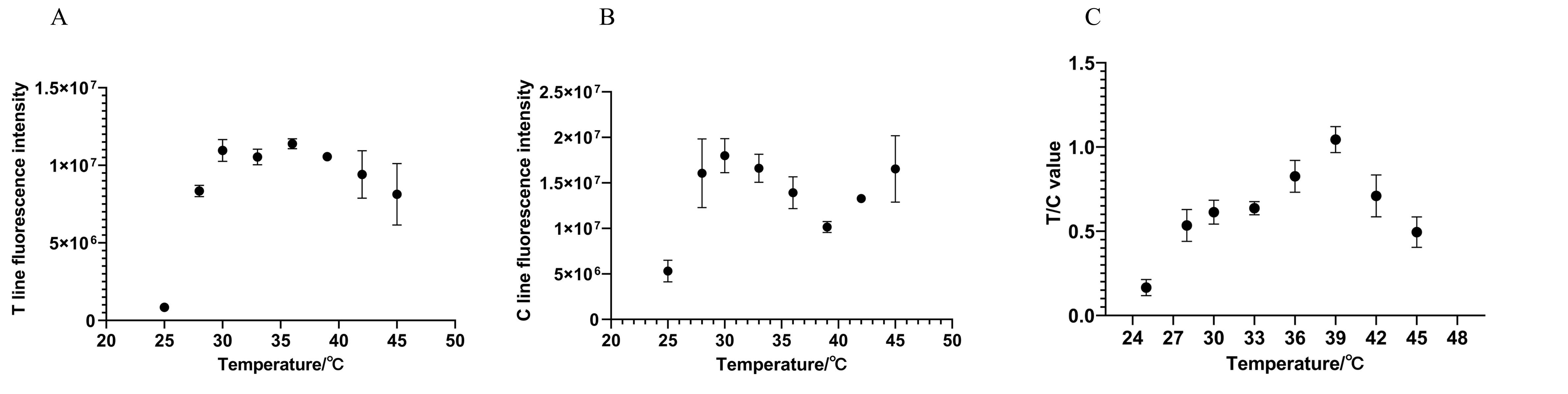

Supplement: Supplementary 3 — Figure 3: optimal temperature screening for RPA detection system. [file 3680778.f3.jpg]

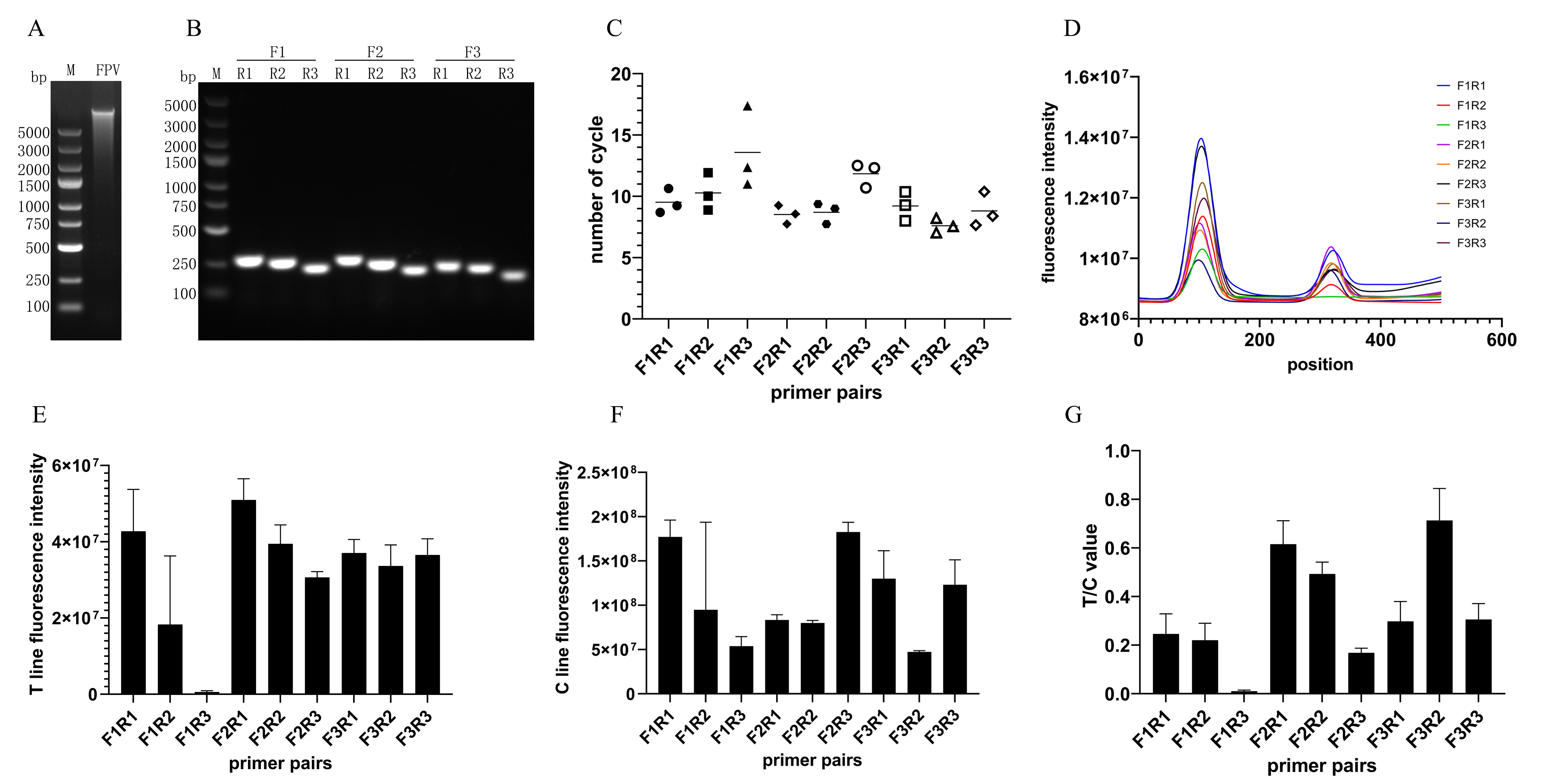

Supplement: Supplementary 5 — Figure 5: results of FPV nucleic acid extraction and primer screening. [file 3680778.f5.jpg]

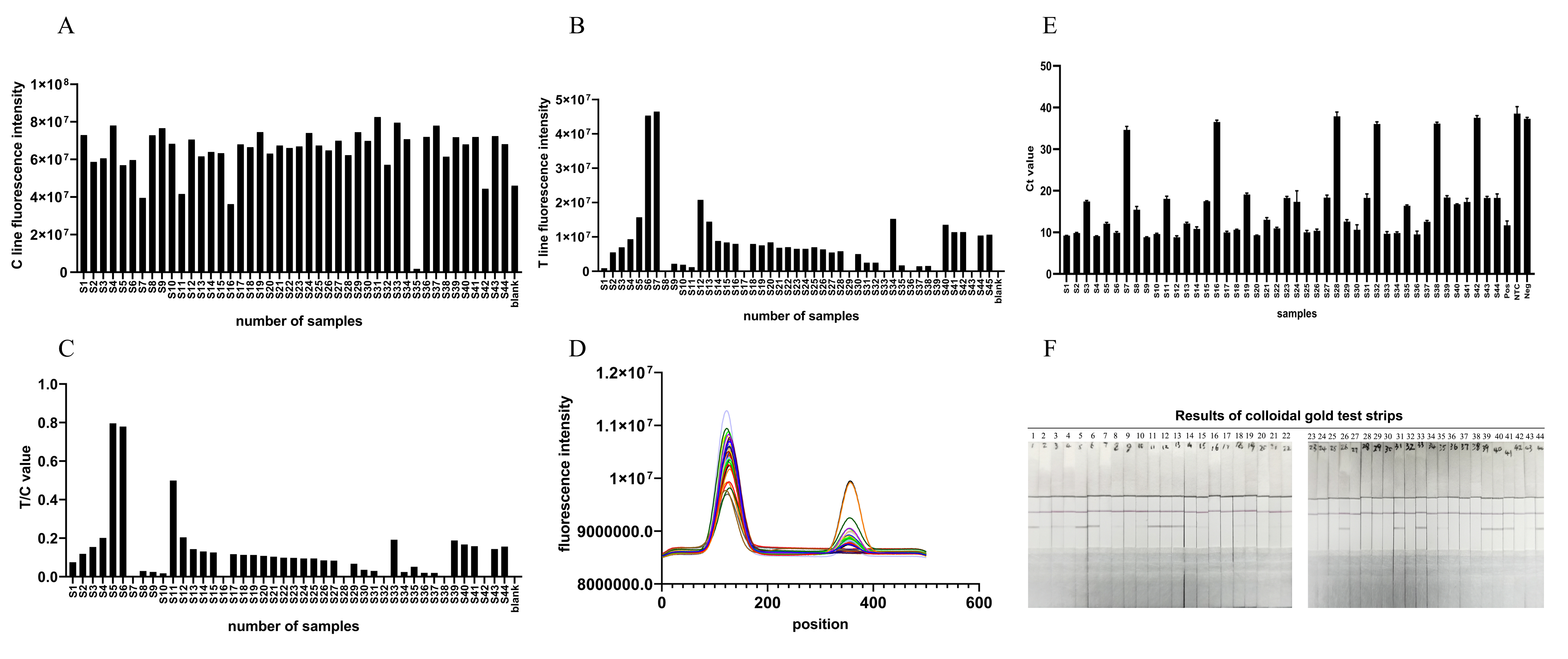

Supplement: Supplementary 6 — Figure 6: the three assays were utilized to examine a total of 44 clinical samples. [file 3680778.f6.png]
