# Supplementary material for: Development and Application of an RPA-Based Rapid Point-of-Care Testing (POCT) Method for the Detection of Feline Panleukopenia Virus
Source: Transbound Emerg Dis. 2024 Aug 24;2024:3680778. doi: 10.1155/2024/3680778 (PMC12016765; doi:10.1155/2024/3680778)
Supplement: Supplementary 4 — Figure 4: original images for gels. [file 3680778.f4.pdf]

Different primers combination for PCR amplification, the amplified fragments were visualized by agarose gel electrophoresis.

Original Images for Gels

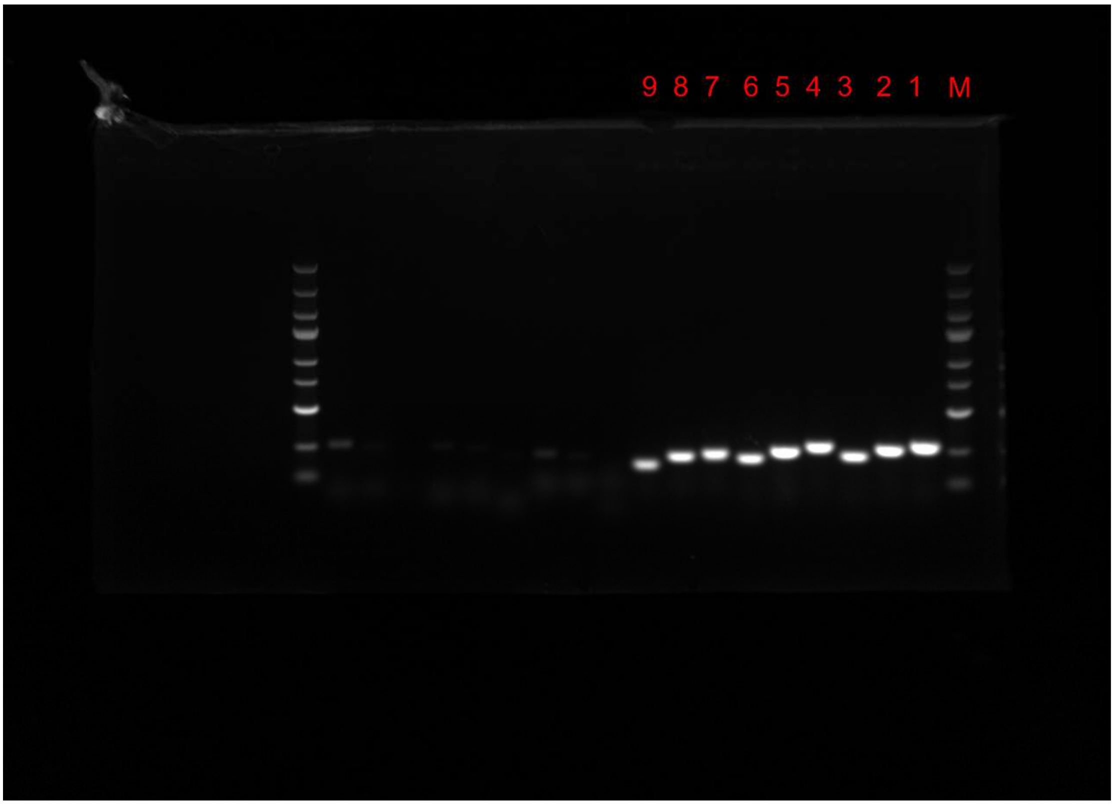

M: Nucleic acid marker.

line 1: PCR amplification results of F1R1 primer pairs.

line 2: PCR amplification results of F1R2 primer pairs.

line 3: PCR amplification results of F1R3 primer pairs.

line 4: PCR amplification results of F2R1 primer pairs.

line 5: PCR amplification results of F2R2 primer pairs.

line 6: PCR amplification results of F2R3 primer pairs.

line 7: PCR amplification results of F3R1 primer pairs.

line 8: PCR amplification results of F3R2 primer pairs.

line 9: PCR amplification results of F3R3 primer pairs.

Results of FPV nucleic acid extraction by TIANamp Virus DNA Kit.

Original Images for Gels

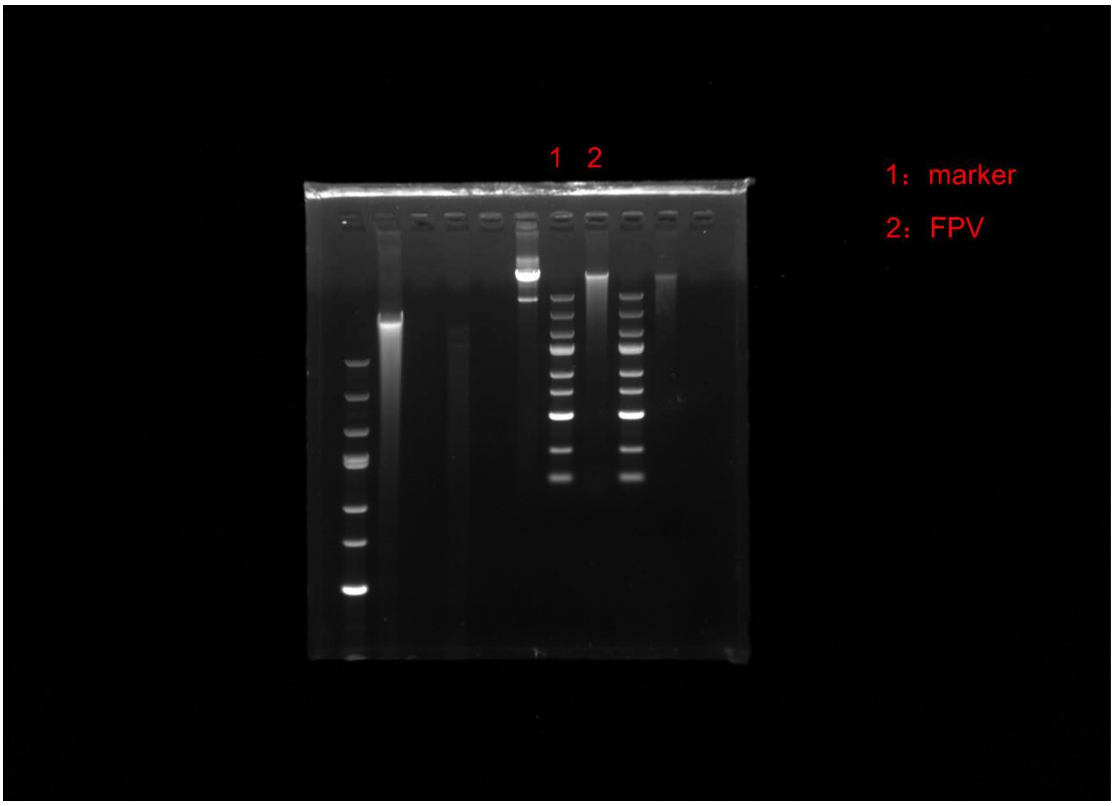

line 1: Nucleic acid marker.

line 2: FPV nucleic acid.
